# The Nitrogen-Removal Efficiency of a Novel High-Efficiency Salt-Tolerant Aerobic Denitrifier, *Halomonas Alkaliphile* HRL-9, Isolated from a Seawater Biofilter

**DOI:** 10.3390/ijerph16224451

**Published:** 2019-11-13

**Authors:** Jilong Ren, Chenzheng Wei, Hongjing Ma, Mingyun Dai, Jize Fan, Ying Liu, Yinghai Wu, Rui Han

**Affiliations:** 1 School of Marine Technology and Environment, Dalian Ocean University, Dalian 116023, China; renjilong@163.com (J.R.); weicz515@163.com (C.W.); hongjingm96@163.com (H.M.); dmingyun@hotmail.com (M.D.); fanjizemail@sina.com (J.F.); yingliu@dlou.edu.cn (Y.L.); 2 Key Laboratory of Environment Controlled Aquaculture, Ministry of Education, Dalian Ocean University, 52 Heishijiao Street, Dalian 116023, China; 3 College of Marine and Civil Engineering, Dalian Ocean University, Dalian 116023, China; wuyinghai@dlou.edu.cn; 4 South China Institute of Environmental Science, Ministry of Ecology and Environment, Guangzhou 510655, China

**Keywords:** aerobic denitrification, nitrogen-removal, salt-tolerant, *napA*, *narG*

## Abstract

Aerobic denitrification microbes have great potential to solve the problem of NO_3_^−^-N accumulation in industrialized recirculating aquaculture systems (RASs). A novel salt-tolerant aerobic denitrifier was isolated from a marine recirculating aquaculture system (RAS) and identified as *Halomonas alkaliphile* HRL-9. Its aerobic denitrification performance in different dissolved oxygen concentrations, temperatures, and C/N ratios was studied. Investigations into nitrogen balance and nitrate reductase genes (*napA* and *narG*) were also carried out. The results showed that the optimal conditions for nitrate removal were temperature of 30 °C, a shaking speed of 150 rpm, and a C/N ratio of 10. For nitrate nitrogen (NO_3_^−^-N) (initial concentration 101.8 mg·L^−1^), the sole nitrogen source of the growth of HRL-9, the maximum NO_3_^−^-N removal efficiency reached 98.0% after 24 h and the maximum total nitrogen removal efficiency was 77.3% after 48 h. Nitrogen balance analysis showed that 21.7% of NO_3_^−^-N was converted into intracellular nitrogen, 3.3% of NO_3_^−^-N was converted into other nitrification products (i.e., nitrous nitrogen, ammonium nitrogen, and organic nitrogen), and 74.5% of NO_3_^−^-N might be converted to gaseous products. The identification of functional genes confirmed the existence of the *napA* gene in strain HRL-9, but no *narG* gene was found. These results confirm that the aerobic denitrification strain, *Halomonas alkaliphile* HRL-9, which has excellent aerobic denitrification abilities, can also help us understand the microbiological mechanism and transformation pathway of aerobic denitrification in RASs.

## 1. Introduction

In recent years, the aquaculture industry has developed rapidly and provided abundant aquatic resources for human beings. Nevertheless, this industry also introduced serious environmental problems [1,2], such as eutrophication in receiving water. Recirculating aquaculture systems (RASs) are considered an important development trend in the aquacultural industry because of their efficient, environmentally friendly, and systemic controllability and high product quality [3]. The treatment and reuse of aquaculture wastewater are the key processes needed for the healthy growth of cultured fish and reducing pollutant emissions. Reducing nitrogen elements and other contaminants from aquaculture water in RASs is an important technical challenge, which has attracted considerable attention in recent years [2].

A biofilter system could effectively remove excessive ammonium nitrogen (NH_4_^+^-N) and nitrous nitrogen (NO_2_^−^-N) from RASs, which have proven harmful to the farmed organisms [4]. The microbes attached to biofilters played an important role in the nitrification process and converted NH_4_^+^-N to nitrate nitrogen (NO_3_^−^-N) through NO_2_^−^-N [5]. Nevertheless, more and more studies have found that the accumulation of NO_3_^−^-N, to a certain extent, could cause serious harm to cultured organisms [6,7,8,9]. Denitrification was expected to solve the problem of nitrate accumulation. NO_3_^−^-N and NO_2_^−^-N could be converted into nitrogen by denitrifiers under anaerobic or anoxic conditions in traditional denitrification theory; four main steps happen in sequence: NO_3_^−^-N→NO_2_^−^-N→NO→N_2_O→N_2_ [10,11]. Four key reductases, nitrate reductase (*Nap*), nitrite reductase (*NirS* or *NirK*), nitric oxide reductase (*Nor*), and nitrous oxide reductase (N_2_OR), correspond to these four steps, respectively [12]. Traditional theory holds that the denitrification process is sensitive to dissolved oxygen (DO) concentrations [13,14]. Nevertheless, RASs need to maintain a high DO to ensure a high-density culture. The anaerobic or anoxic treatment used in the RASs must be followed by an aeration process, which will lead to an increase of costs.

The founding of aerobic denitrifying (i.e., removing NO_3_^−^-N under aerobic conditions by aerobic microbes), offers a possible economic solution to nitrate accumulation in RASs. Since the aerobic denitrifier, *Thiosphera pantotropha*, was first isolated and purified in 1983 [15], more and more studies have found that denitrification can also occur in an aerobic environment [16,17]. In recent years, several aerobic denitrifiers were screened: *Paracoccus versutus* LYM [18], *Paracoccus denitrificans* Strain ISTOD1 [19], *Klebsiella pneumonia* [20], and *Marinobacter* strain NNA5 [21]. The gene *napA*, encoding a periplasmic nitrate reductase, was important for aerobic denitrifiers to remove nitrate under aerobic conditions [14], while the *narG* gene, encoding a membrane-bound reductase, was responsible for bacterial respiration and denitrification under anaerobic conditions [22]. So far, the reported aerobic denitrifiers have mainly been isolated from soils and freshwater lakes. The capacities of the aerobic denitrification of such strains in high salinity (>1%) environments have been greatly limited because high salinity causes a sharp increase in cell osmotic pressure, which produces changes in cellular metabolism and inhibits the enzymatic activity of microbial cells [23]. There were only a few reports about aerobic denitrifiers treating salty wastewater (e.g., *Halomonas campisalis* ha3 [24] and *Marinobacter sp.* F6 [25]), which are limited in high salinity environments. Therefore, it is of scientific and practical significance to separate efficient and salt-tolerant aerobic denitrifiers and investigate the performance of aerobic denitrification in high salinity conditions to solve seawater nitrogen pollution, especially nitrate accumulation.

In this study, a novel highly salt-tolerant (3%) aerobic denitrifier, *Halomonas alkaliphile* HRL-9, was isolated from lab-scale seawater RASs (Dalian, China). The bacterial phenotype, the influence of three important factors (temperature, rotation speed, and C/N ratio), a nitrogen balance analysis, and the functional genes responsible for aerobic denitrification were investigated. We aimed to elucidate the influence of important factors on the aerobic denitrification performance, pathways, and functional genes of HRL-9. This study may contribute to understanding the microbiological mechanisms of aerobic denitrification in RASs.

## 2. Materials and Methods 

### 2.1. Sampling and Media

The samples were obtained from lab-scale seawater RASs of the Dalian Ocean University (Dalian, China). A denitrification medium (DM) was used for bacterial isolation and single-factor affecting experiments. Its components were as follows (per liter): Na_2_HPO_4_ (7.90 g), KH_2_PO_4_ (4.50 g), MgSO_4_ (0.10 g), K_2_HPO_4_ (4.78 g), NaCl (30 g), KNO_3_ (0.72 g), Sodium acetate (3.42 g), and a trace element solution (3.0 mL) [14]. The trace element solution contained (per liter): ZnSO_4_·7H_2_O (3.0 g), H_3_BO_3_ (1.12 g), CaCl_2_ (0.6 g), FeSO_4_·7H_2_O (0.3 g), CuSO_4_·5H_2_O (1.57 g), MgSO_4_·7H_2_O (3.0 g), and EDTA (25 g). The agar plates were prepared by DM containing 20 g agar per liter. A Luria–Bertani (LB) medium was used for bacterial enrichment and to extract the genomic DNA of the isolated strain, HRL-9 (per liter): Agar (20 g), Yeast extract (10 g), NaCl (30 g), pH 7.00. All media were autoclaved at 121 °C for 90 min prior to use (MLS-3781L-PC, Panasonic, Japan).

### 2.2. Strain Isolation

The biological filter samples were taken from the RASs and washed with sterile water. The biofilm was scraped off with a sterilizing blade and gradiently diluted to 10^−2^–10^−7^ with sterile saline. The samples were uniformly plated on agar plates and cultured at 30 °C for 72 h. The monoclonal colonies were inoculated in DM for 48 h. The purified monoclonal strains were stored at −80 °C with 20% glycerol. A total of 21 strains with aerobic denitrification capacity were obtained from this experiment. The isolates were streaked onto a DM medium and incubated for 48 h at 120 rpm and 30 °C, followed by a test of their denitrification ability. Strain HRL-9 had an excellent removal rate of 93.4% for the nitrate in 24 h and was selected for further analyses.

### 2.3. SEM Analysis and Identification of Strain HRL-9

SEM: 2 mL of the bacterial solution was centrifuged at 10,000 rpm for 10 min. The cells were fixed in 3% glutaraldehyde solution for 24 h, and then the morphology of the strain cells was observed by SEM. The genomic DNA of the isolated bacterium HRL-9 was extracted with a TIAamp Bacteria DNA Kit (TIANGEN, China) after cultivation in an LB medium for 24 h. DNA extracts were stored at −20 °C. The amplification of the DNA was performed by polymerase chain reaction (PCR) with 16S universal primers (27F: 5′-AGA GTT TGA TCC TGG CTC AG-3′, 1492R: 5′-GGT TAC CTT GTT ACG ACT T-3′), which were designed by Sangon Biocompany (Shanghai, China). The PCR was performed under the following conditions: a denaturing step of 90 °C for 5 min, followed by 35 cycles of denaturing for 60 s at 95 °C, annealing for 60 s at 55 °C, and a final extension at 72 °C for 5 min. The 16S rDNA gene was compared with that of other bacteria by way of BLAST (https://blast.ncbi.nlm.nih.gov/Blast.cgi). A phylogenetic tree using neighbor-joining method was built by MEGA version 6 (Tamura, Stecher, Peterson, Filipski, and Kumar 2013).

### 2.4. Single-Factor Affecting Aerobic Denitrification

The denitrification capability of the HRL-9 strain was further determined under different conditions, including DO, C/N, and temperature. In the C/N ratio experiments, different amounts of sodium acetate were added with a constant NO_3_^−^-N concentration of 100 mg·L^−1^ to make the ratios of organic carbon and nitrate 3, 5, 10, 15, and 20. The strain was cultivated at 120 rpm and 30 °C for 48 h. In the DO experiments, according to previous reports [16,19] and the results of pre-experiments, the HRL-9 strain was cultivated under shaking speeds of 90 rpm, 120 rpm, and 150 rpm for different concentrations of DO (initial DO concentration: 5.59 ± 0.1 mg·L^−1^, 6.97 ± 0.18 mg·L^−1^, 7.48 ± 0.39 mg·L^−1^). The strain was cultivated at C/N 10 and 30 °C for 48 h. In the temperature experiments, the culture temperature was changed to 20 °C, 30 °C, and 40 °C. The strain was cultivated at 120 rpm and C/N 10 for 48 h. In all single-factor experiments, the strain HRL-9 was inoculated into a 100 mL vial bottle containing 50 mL of a DM medium (initial NO_3_^−^-N 100 mg·L^−1^). The samples were taken at 4 h intervals to measure the growth curve (OD 600), total organic carbon (TOC), NO_3_^−^-N, NO_2_^−^-N, NH_4_^+^-N, and total nitrogen (TN), respectively. All experiments were carried out in triplicate.

### 2.5. Estimation of Aerobic Denitrification Ability of Strain HRL-9

According to the single-factor experiment, strain HRL-9 was inoculated into a 100 mL vial bottle containing 50 mL of the DM medium (initial NO_3_^−^-N 100 mg·L^−1^) at optimal conditions (150 rpm, 30 °C, C/N 10). The aerobic denitrification ability was assessed after 48 h. The vial bottle was sealed with sterile breathable sealing membranes. The samples were taken periodically to determine the growth curve (OD600), TOC, NO_3_^−^-N, NO_2_^−^-N, NH_4_^+^-N, and TN. All experiments were carried out in triplicate.

### 2.6. Nitrogen Balance Analysis

Nitrogen balance analysis was used to investigate the nitrogen conversion pathway by the aerobic denitrifying bacteria HRL-9. The total removal rate of the nitrogen by strain HRL-9 was then determined. The culture collected by the centrifuge tube was placed in an ice box, to ensure the temperature did not rise during sonication, and was then disrupted by an ultrasonic cell crusher (JY92-IIN, Ningbo Scientz Biotechnology Co., Ltd., Ningbo, China). Samples were sonicated to ensure the final TN contains intracellular TN. The ultrasonication process was as follows: the ultrasonic worked in one cycle consisting of a 4 s pulse and a 6 s pause. Each sample underwent 90 cycles of sonication using a 6 mm ultrasonic probe. The bacterial solution obtained by ultrasonication was centrifuged at 8000 rpm for 10 min. Supernatants were filtered using 0.22 μm membrane filters, and the filtrates were used to detect the contents of the final soluble TN, NO_3_^−^-N, NO_2_^−^-N, and NH_4_^+^-N. The data calculation methods are as follows:

% Intracellular-N = (final TN − final soluble TN)/initial TN × 100%(1)

Final organic-N = final soluble TN − (final NH_4_^+^-N) − (final NO_3_^−^-N) − (final NO_2_^−^-N) (2)

% N removal = [(initial TN) − (final NH4^+^-N) − (final NO_3_^−^-N) − (final NO_2_^−^-N) − (final organic-N) − (final intracellular-N)]/initial TN × 100%. (3)

### 2.7. Amplification of napA and narG Gene

HRL-9 was inoculated in an LB medium for 24 h. Then, the bacterial suspensions were used for genomic DNA extraction with a bacterial DNA kit (TIAamp Bacteria DNA Kit, TIANGEN). The genomic DNA was used as a template for nitrate reductase gene (*napA* and *narG*) amplification. The PCR amplification of the *napA* gene was conducted using primer F: 5′-CAGCTCTATGCCGATCCGAG-3′ and R: 5′-CAGCCCCTCTTCGTTCATGT-3′; The PCR amplification of *narG* gene was conducted using a primer according to Roussel-Delif et al. [26] F: 5′-GAYATGCAYCCGTT-3′, R: 5′-AYCCARTCRTTRTC-3′. The PCR protocol consisted of the following steps (Biometra GmbH, Germany): pre-denaturation at 94 °C for 5 min, 35 cycles of denaturation at 94 °C for 30 s, annealing for 30 s at 65 °C for *napA* and at 50 °C for 30 s for *narG*, and a final extension at 72 °C for 7 min. The PCR products were electrophoresed on a 2% agarose gel using an electrophoresis apparatus.

### 2.8. Analytical Methods

The concentration of DO was determined by a YSI multi-parameter water quality measuring instrument (556MPS, USA). The bacterial growth (OD600) was monitored by measuring the optical density (OD) at 600 nm using a spectrophotometer (10s uv-vis, Thermo Fisher Genesys, Waltham, MA, USA). Concentrations of NH_4_^+^-N, NO_3_^−^-N, NO_2_^−^-N, and TN were determined with a continuous-flow auto-analyzer (AA3, Seal Analytical Inc, Southampton, UK). The TOC concentration was determined using a TOC-L analyzer (Shimadzu, Kyoto, Japan). A one-way analysis of variance (ANOVA) was applied to assess the difference of the removal efficiencies under different conditions using SPSS Statistics version 24.0 (IBM, Armonk, NY, USA). Values of *p* < 0.05 were taken to show statistical significance at a 95.0% confidence level.

## 3. Results

### 3.1. Identification of Strain HRL-9

**Figure 1:**
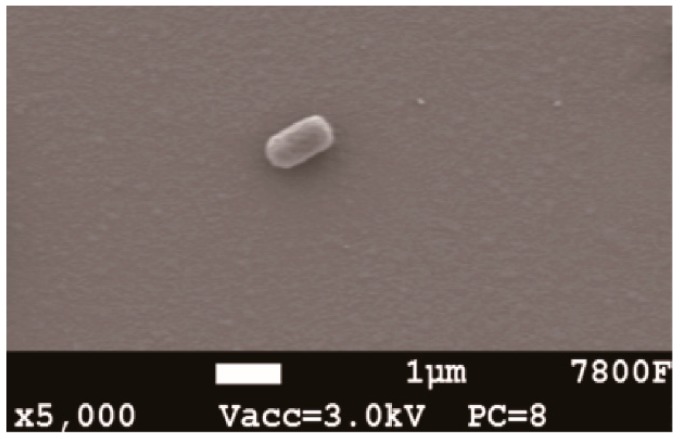
SEM images of aerobic denitrifier *Halomonas alkaliphile* HRL-9 after 48 h cultivation.

**Figure 2:**
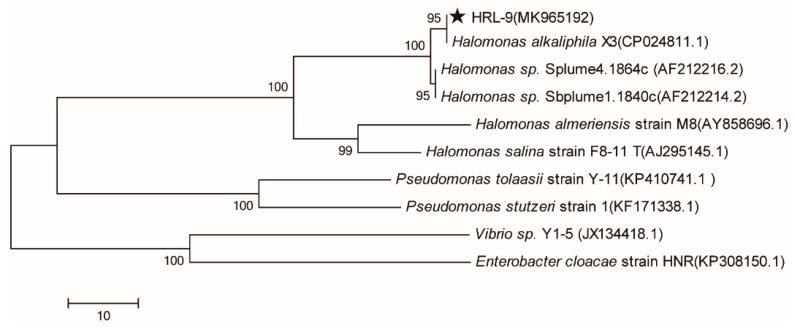
The phylogenetic tree of *Halomonas alkaliphile* HRL-9 derived from a neighbor-joining analysis of partial 16S rDNA sequences. Strain HRL-9 was labeled with five-pointed star.

The mature colony of strain HRL-9 was yellow, circular shaped, slightly bulged, smooth at the edges, and opaque on the agar plates. Strain HRL-9 was Gram-negative. The SEM image shows that the strain HRL-9 contains shorts rod with a width of about 0.4–0.6 μm and a length of 1.2–1.3 μm (Figure 1). A 1437 bp fragment of 16S rDNA was obtained by PCR and sequenced; it was then deposited in GenBank with the accession number MK965192. The BLAST results assigned strain HRL-9 to the genus Halomonas sp, which showed a high similarity to Halomonas alkaliphila X3 (Figure 1). Therefore, strain HRL-9 was identified as *Halomonas alkaliphile* HRL-9. A phylogenetic tree was constructed based on 16S rDNA gene sequences of the isolate and some other phylogenetically related strains (Figure 2).

### 3.2. The Influence of Shaking Speed, Temperature and C/N Ratio on Aerobic Denitrification

#### 3.2.1. Effect of Shaking Speed

**Figure 3:**
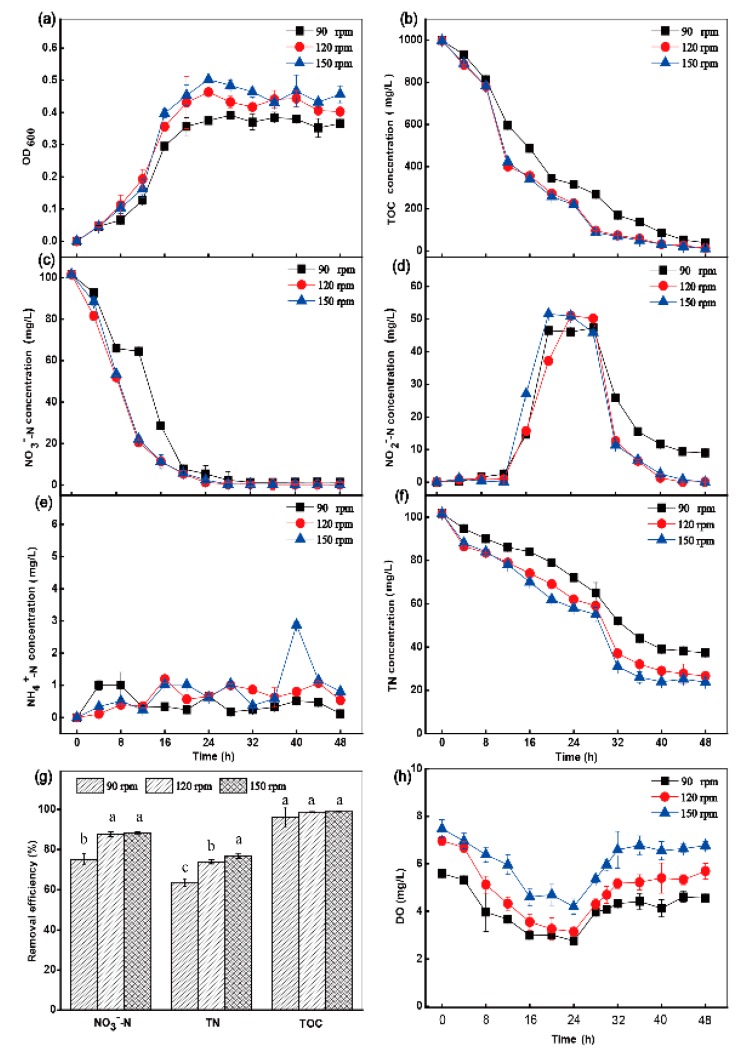
The effect of shaking speed on OD600 (**a**), TOC (**b**), NO_3_^−^-N (**c**), NO_2_^−^-N (**d**), NH_4_^+^-N (**e**), TN (**f**) aerobic environments by *Halomonas alkaliphile* HRL-9. The removal efficiencies of NO_3_^−^-N, TN, and TOC in 48 h at different shaking speed (**g**). The influence of shaking speed on DO (**h**). Values are the means ± SD for triplicates. The letters (a, b and c above the columns in Figure 3g) were used to show the significant difference (*p* < 0.05).

The experimental results showed that the growth rate of HRL-9 was slow at a shaking speed of 90 rpm (Figure 3a). At a shaking speed of 90 rpm, the initial concentration of DO was 5.59 ± 0.10 mg·L^−1^, decreased to 2.76 mg·L^−1^ in 24 h, and then finally recovered to 4.55 mg·L^−1^ in 48 h (Figure 3h). In this case, the TOC utilization rate was 96.1% (Figure 3b), the removal rate of NO_3_^−^-N was 94.7% at 48 h, the NO_2_^−^-N concentration was 8.98 mg·L^−1^ at 48 h (Figure 3d), and the final removal rate of TN was 63.3% (Figure 3f,g). At a shaking speed of 120 rpm (the initial DO concentration was 6.97 ± 0.18 mg·L^−1^) and 150 rpm (the initial DO concentration was 7.48 ± 0.39 mg·L^−1^), the DO concentration decreased to 3.15 mg·L^−1^ and 4.19 mg·L^−1^ in 24 h, respectively (Figure 3h). Simultaneously, NO_3_^−^-N was almost completely removed at 48 h with a removal efficiency of 99.8% (Figure 3c). The removal efficiency of TN at a shaking speed of 120 rpm and 150 rpm was 73.83% and 76.58% at 48 h, respectively (Figure 3f,g). There was no accumulation of NO_2_^−^-N in the reaction system (Figure 3d). Different concentrations of NH_4_^+^-N were produced under three different shaking conditions (Figure 3e). DO concentration changed too significantly during batch cultivation (Figure 3h).

#### 3.2.2. Effect of Temperature

**Figure 4:**
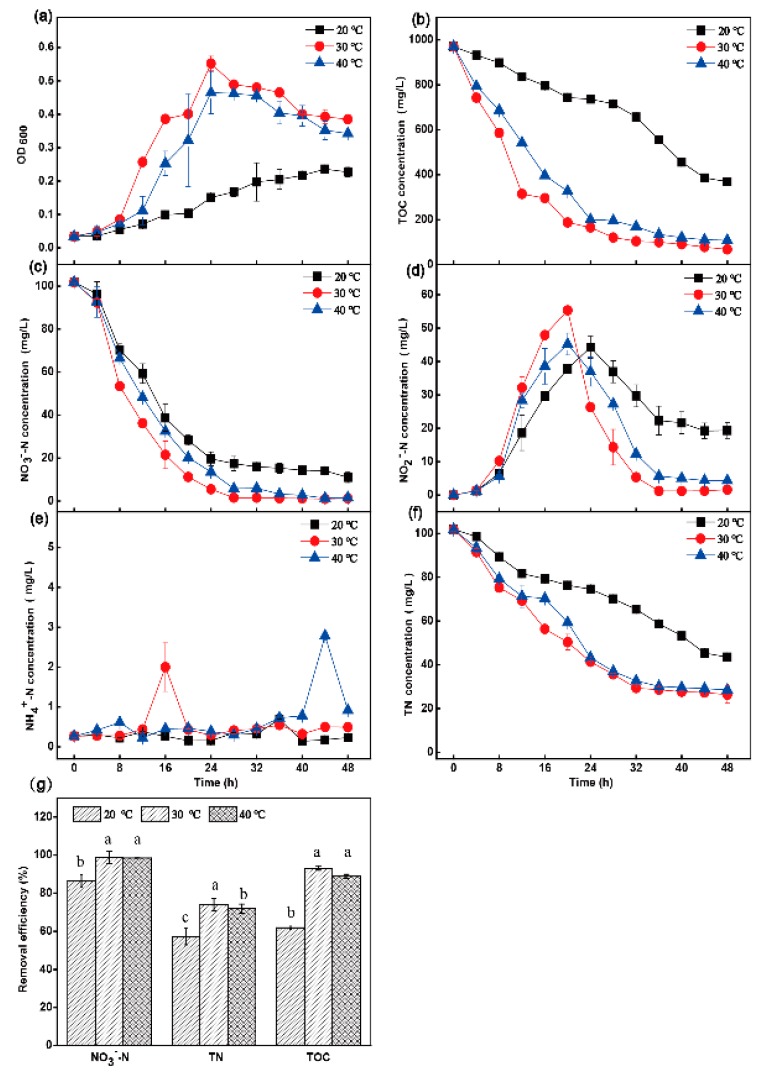
The effect of temperature on OD600 (**a**), TOC (**b**), NO_3_^−^-N (**c**), NO_2_^−^-N (**d**), NH_4_^+^-N (**e**), and TN (**f**) aerobic environments by *Halomonas alkaliphile* HRL-9. The removal efficiencies of NO_3_^−^-N, TN, and TOC in 48 h at different shaking speed (**g**). Values are the means ± SD for triplicates. The letters (a, b and c above the columns in Figure 4g) were used to show the significant difference (*p* < 0.05).

The results showed that the strain grew slowly at 20 °C, and the TOC utilization rate was 62.0% (Figure 4a,b); the removal rate of NO_3_^−^-N reached 89.2% at 48 h, and the removal rate of TN was only 57.3% (Figure 4f,g). In addition, the accumulation of NO_2_^−^-N was serious. The NO_2_^−^-N concentration was 44.3 mg·L^−1^ at 24 h, and the NO_2_^−^-N concentration still reached 19.3 mg·L^−1^ at 48 h. At 40 °C, the removal rate of NO_3_^−^-N was 94.3% at 48 h (Figure 4c), and the final removal rate of TN reached 72.0% (Figure 4f,g). There was a slight accumulation of NO_2_^−^-N (4.35 mg·L^−1^) at 40 °C (Figure 4d). At 30 °C, the aerobic denitrifying bacteria grew faster than when the temperature was 20 °C and 40 °C (Figure 4a), the removal rate of NO_3_^−^-N was 98.8% at 48 h (Figure 4c), the final removal rate of TN was 74.2% (Figure 4f,g), and there was no NO_2_^−^-N accumulation at 48 h (Figure 4d). There was a small amount of ammonia nitrogen production under the three different conditions (Figure 4e).

#### 3.2.3. Effect of C/N

**Figure 5:**
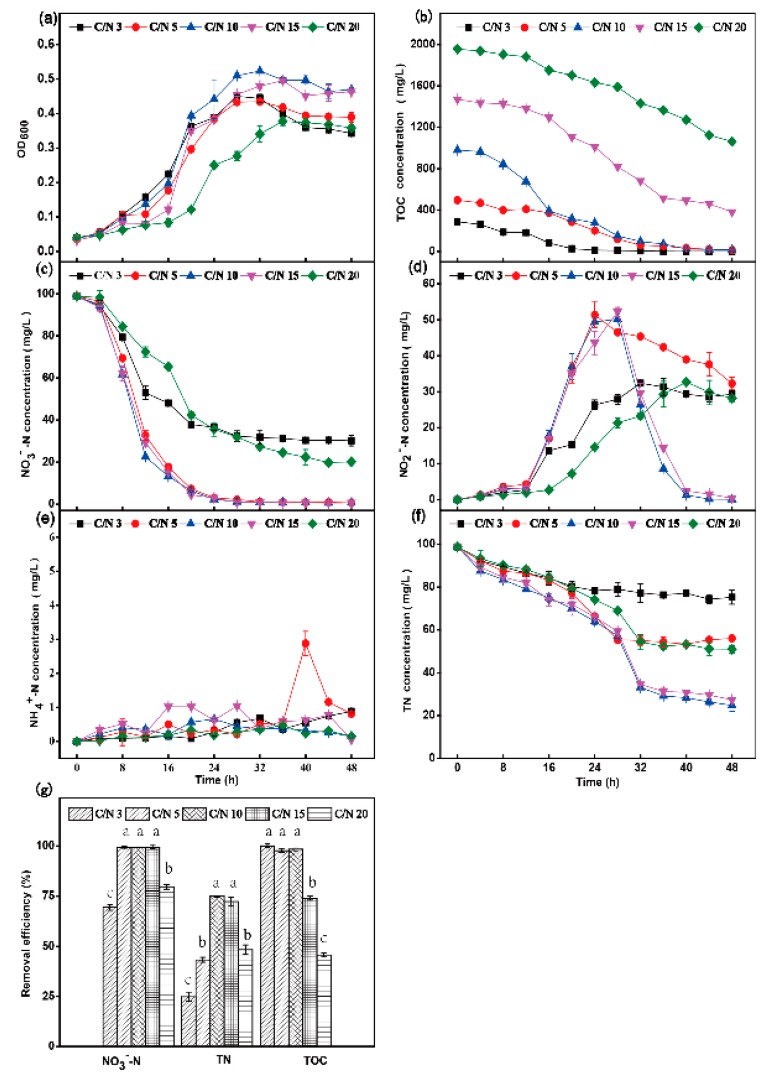
The effect of C/N on OD600 (**a**), TOC (**b**), NO_3_^−^-N (**c**), NO_2_^−^-N (**d**), NH_4_^+^-N (**e**), and TN (**f**) aerobic by *Halomonas alkaliphile* HRL-9. Removal efficiencies of NO_3_^−^-N, TN, and TOC in 48 h at different shaking speeds (**g**). Values are the means ± SD for triplicates. The letters (a, b and c above the columns in Figure 5g) were used to show the significant difference (*p* < 0.05).

In order to investigate the effect of the C/N ratio on the aerobic denitrification capacity of strain HRL-9, the strain was inoculated into a DM medium with NO_3_^−^-N (initial concentration 98.8 mg·L^−1^) as the sole nitrogen source. The experimental results are shown in Figure 5. At C/N ratios of 3 and 5, the OD600 decreased after the strain was cultured for 24 h, and the logarithmic growth period was shorter than that at a C/N ratio of 10 (Figure 5a). At a C/N ratio of 3, the removal rate of NO_3_^−^-N reached 69.5% at 48 h (Figure 5c). The concentration of NO_2_^−^-N was 29.3 mg·L^−1^ at 48 h, and the final removal rate of TN reached 23.8% (Figure 5g). At a C/N ratio of 5, the removal rate of NO_3_^−^-N was 99.3% at 48 h (Figure 5c). The concentration of NO_2_^−^-N was 32.3 mg·L^−1^, and the final removal rate of TN reached 43.3% (Figure 5d,g). The removal rates of NO_3_^−^-N at C/N ratio 10 and 15 were 99.3% and 98.9% (Figure 5c), respectively. There was no NO_2_^−^-N accumulation in the system at 48 h, and the concentrations of NO_2_^−^-N at C/N ratio 10 and 15 were 0.08 mg·L^−1^ and 0.36 mg·L^−1^ (Figure 5d), respectively. The final removal rates of TN were 74.9% and 72.3% (Figure 5g), respectively. At a C/N ratio of 20, the removal rate of NO_3_^−^-N reached 79.6% at 48 h (Figure 5c), the final concentration of NO_2_^−^-N was 28.2 mg·L^−1^ (Figure 5d), and the removal rate of TN reached 48.3% at 48 h (Figure 5g).

### 3.3. Aerobic Denitrification and Nitrogen Balance of Strain HRL-9

**Figure 6:**
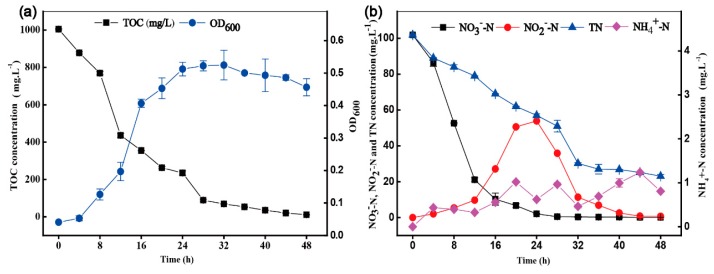
Changes of water quality parameters in denitrification by *Halomonas alkaliphile* HRL-9 in experiment system under optimal condition (150 rpm, 30 °C, C/N 10). TOC, OD600 (**a**); TN, NO_3_^−^-N, NO_2_^−^-N, NH_4_^+^-N (**b**). Values are the means ± SD for triplicates.

HRL-9 grew slowly in 4 h and reached a logarithmic growth phase at 8 h–32 h under optimal conditions (Figure 6). After 32 h, the growth of bacterial growth was negative. The utilization efficiency of TOC was 29.2 mg·L^−1^ from 8 h to 32 h (Figure 6a). The removal efficiency of NO_3_^−^-N reached 98.0% with an initial concentration about 100 mg·L^−1^ at 24 h. The accumulation of NO_2_^−^-N reached 53.9 mg·L^−1^ at 28 h. The accumulated NO_2_^−^-N was almost completely removed at 48 h. The final removal rate of TN reached 77.3% (Figure 6b).

The results of the nitrogen balance analysis are shown in Table 1. During the initial stage of denitrification, NO_3_^−^-N (101.4 ± 0.02 mg·L^−1^) was the sole nitrogen source. The NO_3_^−^-N concentration decreased to 1.24 mg·L^−1^ at 48 h, about 21.7% of NO_3_^−^-N was converted to intracellular nitrogen, 3.3% of NO_3_^−^-N was converted to other nitrification products (NO_2_^−^-N, NH_4_^+^-N and organic nitrogen), and 74.5% of NO_3_^−^-N might be converted to gaseous products.

### 3.4. Amplification of napA and narG Gene

**Figure 7:**
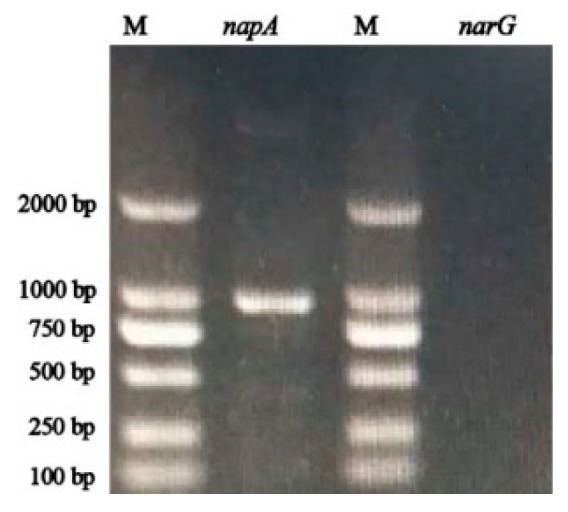
Amplification results of *napA* and *narG* from strain *Halomonas alkaliphile* HRL-9 (M: DL 2000 DNA marker).

The *napA* gene is an essential functional gene for the transformation of nitrate under aerobic conditions [27]. In this study, the *napA* gene of 884 bp products was amplified successfully, while the *narG* gene was not obtained (Figure 7).

## 4. Discussion

Strain HRL-9, isolated from seawater biofilter, was identified as *Halomonas alkaliphile* HRL-9. The genus *Halomonas sp.* includes many strains with the capability of aerobic denitrification; these strains include *Halomonas desiderata sp.* Nov [28], *Halomonas chromatireducens sp.* [29], and *Halomonas campisalis* ha3 [24]. This indicates that *Halomonas sp.* is a common genus with the function of aerobic denitrification. The results of a single-factor experiment showed that different factors (DO, temperature, C/N) had significant effects on the aerobic denitrification performance of the strain HRL-9 (Figure 3, Figure 4 and Figure 5).

At 150 rpm (the initial DO concentration was 7.48 ± 0.39 mg·L^−1^), the performance of the aerobic denitrification capacity of strain HRL-9 was significantly higher than that under other conditions (Figure 3). When the shaking speed was 90 rpm, the removal rate of NO_3_^−^-N was 94.7% at 48 h, and the NO_2_^−^-N concentration was 8.98 mg·L^−1^ at 48 h (Figure 3c,d). However, when the shaking speed was increased to 150 rpm, the removal rate of NO_3_^−^-N was increased to 99.7%, and there was no accumulation of NO_2_^−^-N (Figure 3c,d). The removal efficiency of TN at a shaking speed of 150 rpm was the highest at 48 h (Figure 3g). Therefore, the aerobic denitrification capacity of the strain HRL-9 increased with an increase of the DO concentration, which was similar to the reported results [30,31]. Figure 3h shows that the DO concentration gradually decreases over 24 h. Then, the DO concentration gradually increased at 28 h until it remained stable at 40 h. This may be because the strain HRL-9 presented a relatively high growth rate and consumed a large amount of DO in 24 h (Figure 3a,h). After 28 h, the growth rate of the strain decreased, and the DO concentration gradually recovered (Figure 3a,h). This result was similar to the results of Zhao et al. [16]. Strain HRL-9 performed best at 150 rpm (the initial DO concentration was 7.48 ± 0.39 mg·L^−1^), which also showed a high DO tolerance limit. Zhou et al. [32] also found that *Pseudomonas stutzeriIt* tolerated a high DO level. Therefore, strain HRL-9 has a potential application in the purification process of aquaculture waters, which need to maintain a high DO concentration. Temperature was reported to be an important factor affecting denitrification capacity [14]. In this study, the aerobic denitrification strain HRL-9 had the best denitrification performance and a significantly higher removal rate of nitrogen than other conditions at 30 °C (Figure 4). This result was similar to that of the reported denitrifiers, *P. stutzeri* XL-2 [14], *P. stutzeri* ZF31 [33], and *Halomonas.sp.* 5CRT [34]. This might be due to the effect of temperature on the reductase expression of denitrifying bacteria, which affects the denitrification rate of the strains [14]. Organic carbon played an important role in bacterial cell growth and heterotrophic denitrification [14]. The experimental results showed that the C/N ratio could significantly affect the denitrification efficiency of aerobic denitrifying bacteria HRL-9. At C/N ratios of 3 and 5, the experimental system had a low nitrogen removal efficiency and its nitrite accumulation was severe (Figure 5). Kim et al. [35] found that the electron flow at a low carbon concentration was too low to provide sufficient energy for bacterial cell growth. In this study, the denitrification performance was better at C/N ratios of 10 and 15 than under other conditions. Moreover, the nitrogen removal efficiency was slightly higher at a C/N ratio of 10 than 15. This may be because the microbe needed a carbon source to maintain cell growth and the aerobic denitrification process [14,36]. Interestingly, this study found that nitrogen removal efficiency was significantly reduced at a C/N ratio of 20. It is speculated that the concentration of organic carbon exceeded the requirements of bacteria growth, which inhibited the denitrification rate of HRL-9 [35]. Simultaneously, the different demands for DO in different growth stages of bacteria might lead to slight changes in DO concentration, but the changing trends of DO concentrations between the different treatments were almost the same, and the DO concentration was not the key factor leading to the differences in Figure 5g. These results are similar to the effects of C/N on the denitrification rate of strain XL-2 [14]. In the C/N ratio experiments, the results indicated that HRL-9 has the highest removal efficiency of nitrate at a C/N ratio of 10 (Figure 5g). Simultaneously, the carbon source is almost completely utilized (Figure 5b). Therefore, strain HRL-9 can completely remove nitrate without causing other environmental problems. In the single factor test, the effect of NH_4_^+^-N concentration on the denitrification rate of HRL-9 were produced under different conditions, but no significant difference was shown. This result is similar to the determination of DNRA phenotype of denitrifying bacteria [10]. The change in pH value during cultivation was evaluated (from 7.00 to 7.50). The pH remained stable and rose slightly. The result had minor and similar effect on all experiment treatment groups. Based on the results of the single factor experiments, the optimal conditions for the growth and denitrification of strain HRL-9 are a shaking speed of 150 rpm, a temperature of 30 °C, and a C/N ratio of 10.

HRL-9 had excellent denitrification performance and efficient nitrogen removal efficiency under optimal conditions (Figure 6). Furthermore, the nitrogen balance of strain HRL-9 showed that 21.7% of NO_3_^−^-N was converted into intracellular nitrogen, 3.3% of NO_3_^−^-N was converted into other nitrification products (i.e., nitrous nitrogen (NO_2_^−^-N), ammonium nitrogen (NH_4_^+^-N), and organic nitrogen)), and 74.5% of NO_3_^−^-N might be converted to gaseous products (Table 1). This result indicates that the aerobic denitrification by strain HRL-9 was the predominant fate for nitrate reduction in the experimental system. These results are similar to those of the *Pseudomonas stutzeri* strain ZF31 [33]. The *napA* gene encoded a periplasmic nitrate reductase, which allowed denitrifying bacteria to reduce NO_3_^−^-N under aerobic conditions. This was essential for the transformation of nitrate under aerobic conditions [22,27,37]. The *narG* gene encoded a membrane-bound reductase and is an important functional gene for bacterial respiration and denitrification under anaerobic conditions [38,39]. Functional gene amplification revealed that strain HRL-9 contains the essential gene *napA*, which allowed aerobic denitrifying bacteria to reduce nitrate in an aerobic environment (Figure 7). This result, together with the above experimental results, revealed that *Halomonas alkaliphile* HRL-9 isolated from the seawater RASs has the potential to reduce nitrate in an aerobic and high salt environment.

## 5. Conclusions

In conclusion, a novel salt-tolerant aerobic denitrifier, *Halomonas alkaliphile* HRL-9 was isolated from laboratory seawater RASs of Dalian Ocean University (Dalian, China). Single-factor experiment results showed the highest removal rate of nitrate to appear at a C/N ratio of 10, a temperature of 30 °C, and a shaking speed of 150 rpm. A total of 98.0% of NO_3_^−^-N was removed at 24 h, and 77.3% of TN was removed at 48 h. A nitrogen balance analysis of strain HRL-9 revealed that approximately 74.5% of the initial nitrogen was removed as gas products, and 21.7% of NO_3_^−^-N was converted to intracellular nitrogen. The existence of the *napA* gene, rather than the *narG* gene, was responsible for the excellent aerobic denitrification ability of strain HRL-9. This study revealed that strain HRL-9 could efficiently remove nitrogen from salty wastewater. Strain HRL-9 has the potential to solve the accumulation of nitrate in RAS by immobilization and microbial augmentation. Also, the method of feeding carbon source is important. In addition, this study can help us understand the microbiological mechanism of aerobic denitrification in RASs.

## Figures and Tables

**Table 1:** Nitrogen balance analysis of the aerobic denitrification process of *Halomonas alkaliphile* HRL-9 after 48 h of cultivation under aerobic conditions. Values are the means ± SD for triplicates.

Substance	Initial TN mg·L^−^^1^	Final TN mg·L^−^^1^				Intracellular-N/%	Nitrogen Remove/%
		NO_3_^−^-N	NO_2_^−^-N	NH_4_^+^-N	Organic-N		
Nitrate	101.4 ± 0.02	1.24 ± 0.01	0.10 ± 0.01	0.50 ± 0.10	2.75 ± 0.32	21.7 ± 0.28	≈74.5
